# Mutations in VP0 and 2C Proteins of Duck Hepatitis A Virus Type 3 Attenuate Viral Infection and Virulence

**DOI:** 10.3390/vaccines7030111

**Published:** 2019-09-11

**Authors:** Xingjian Wen, Jinlong Guo, Di Sun, Mingshu Wang, Dian Cao, Anchun Cheng, Dekang Zhu, Mafeng Liu, Xinxin Zhao, Qiao Yang, Shun Chen, Renyong Jia, Ying Wu, Shaqiu Zhang, Sai Mao, Xumin Ou, Xiaoyue Chen, Yanling Yu, Ling Zhang, Yunya Liu, Bin Tian, Leichang Pan, Mujeeb Ur Rehman

**Affiliations:** 1 Institute of Preventive Veterinary Medicine, Sichuan Agricultural University, Chengdu 611130, Sichuan, China; wenxingjian1990@163.com (X.W.); jinlong666guo@163.com (J.G.); sundi0921@126.com (D.S.); mshwang@163.com (M.W.); spzcaodian@163.com (D.C.); zdk24@sicau.edu.cn (D.Z.); liumafengra@163.com (M.L.); xxinzhao@sicau.edu.cn (X.Z.); yangqiao721521@sina.com (Q.Y.); shunchen@sicau.edu.cn (S.C.); cqrc_jry@163.com (R.J.); yingzi_no1@126.com (Y.W.); shaqiu86@hotmail.com (S.Z.); sarrawin@163.com (S.M.); omlx19881130@163.com (X.O.); yanling3525@163.com (Y.Y.); zl97451@126.com (L.Z.); yunnyaaliu@163.com (Y.L.); pl2007@126.com (L.P.); mujeebnasar@yahoo.com (M.U.R.); 2 Research Center of Avian Diseases, College of Veterinary Medicine, Sichuan Agricultural University, Chengdu 611130, Sichuan, China; 3 Key Laboratory of Animal Disease and Human Health of Sichuan Province, Sichuan Agricultural University, Chengdu 611130, Sichuan, China

**Keywords:** duck hepatitis A virus, genetics, pathogenicity, attenuation, hepatic injury, innate immune response, intense inflammation, cytokine

## Abstract

Duck hepatitis A virus (DHAV) is prevalent worldwide and has caused significant economic losses. As the predominant serotype in China, DHAV-3 has become a major challenge to the local duck industry. Here the genetics and pathogenesis of a virulent DHAV-3 strain and its embryo-passaged strain were assessed. There were only two amino acid substitutions (Y164N in VP0 protein and L71I in 2C protein) introduced during the adaptation process. The pathogenicity of these strains was further evaluated in vivo. Clinical signs, gross pathology, and histopathological analysis showed that the embryo-passaged strain was attenuated. Meanwhile, the viral RNA loads were significantly lower in the liver tissues of the ducklings infected with the attenuated strain. As expected, infection with the virulent and attenuated strains led to the activation of different innate immune genes. We suspected that the loss of replication efficiency in ducklings was responsible for the attenuation phenotype of the embryo-passaged strain. In addition, different innate immune responses in the liver of ducklings were at least partly responsible for the differential infectivity phenotype. These findings provide new insights into the genetics and pathogenesis of DHAV-3, which may aid the development of new vaccines and the implementation of immunization strategies.

## 1. Introduction

Duck viral hepatitis (DVH) is a highly fatal and acute disease that has resulted in significant losses to young ducklings worldwide. DVH is caused by divergent viruses, which is a concern for global duck production [1]. Duck hepatitis A virus (DHAV), which consists of three serotypes (DHAV-1, -2, and -3), is the primary causative pathogen of this disease. Despite the application over several decades, the effectiveness of DHAV-1 vaccines has declined in recent years [2,3]. DHAV-1 variants and DHAV-3 outbreaks are the major reasons for the reduced effectiveness of DHAV-1 vaccines against DVH [4]. Furthermore, a recent epidemiological survey reported that DHAV-3 has become the predominant serotype in China [2]. Therefore, the development of an effective attenuated vaccine based on the DHAV-3 isolate emerging in China is urgently needed.

Pathogenic DHAV replication in various tissues and organs leads to severe DVH, which causes high mortality among young ducklings [1]. Notably, DHAV can replicate and persist at high levels in the liver tissues of ducklings, resulting in hepatic injury [5,6,7]. Previous in vitro studies have correlated different immunomodulatory properties to the pathogenicity of DHAV-1 in ducklings [8,9,10]. The innate immune system detects and responds to viral infections through pattern recognition receptors (PRRs), resulting in the production of diverse cytokines, such as type I interferons (IFN-α and -β), type II interferon (IFN-γ), tumor necrosis factor alpha (TNF-α), and various interleukins (ILs), which have different immunoregulatory functions during the immune response to limit viral propagation. However, the molecular mechanisms regulating the pathogenicity of DHAV have not yet been fully elucidated. Furthermore, recent evidence has suggested that the innate immune responses to viral infection may also serve as drivers of autoimmunity and play various roles in the pathogenesis of hepatotropic viruses [11,12,13,14]. Therefore, it is critical to understand how the induction of cytokines and subsequent inflammation are regulated in response to DHAV-3 infection. Recent data suggest that the pathogenic properties of DHAV-3 are similar to those of classical DHAV-1 [15,16]. However, no study has yet clarified the pathophysiological and immunological mechanisms of DHAV-3 isolates with varying degrees of pathogenicity.

In this study, the complete genomes of a pathogenic DHAV-3 strain and its embryo-adapted strain were determined. Comparative genomic analysis revealed that only two amino acid substitutions are produced during the embryo adaptation process of the parental strain. In addition, an in vivo study showed that the embryo-adapted strain was attenuated and non-virulent to 7-day-old ducklings. The viral RNA load was significantly lower in the livers of ducklings infected with the attenuated strain than those inoculated with the virulent strain, which mainly coincides with the gross pathology caused by these strains. Furthermore, the virulent parental strain and its attenuated strain were found to induce different magnitudes of histopathologic lesions and the innate immune responses in the livers of infected ducklings. Taken together, these results demonstrate significant differences in the replication capacity, pathogenicity of DHAV-3, and activation of host responses by pathogenic vs. non-virulent DHAV-3 strains. The attenuated strain has a reduced replication capacity and can reactivate a well-regulated inflammatory response in the liver tissues of infected ducklings.

## 2. Materials and Methods

### 2.1. Animal and Ethics Statement

Specific-pathogen-free duck eggs and 7-day-old healthy Cherry Valley ducklings were obtained from the Harbin Veterinary Research Institute (Harbin, China) and a farm operated by Sichuan Agricultural University (Sichuan, China), respectively. Over the entire experimental period, the experimental ducklings were maintained in an isolation environment and provided with food and water ad libitum. All of the Cherry Valley ducklings were healthy and negative for DHAV antibodies and all major duck infectious pathogens, as confirmed by a previously established quantitative polymerase chain reaction (PCR) technique [17,18,19]. The experimental animal protocol was approved by the Ethical and Animal Welfare Committee of Sichuan Agriculture University and conducted in accordance with the Chinese version of the Guide for the Care and Use of Laboratory Animals. All efforts were made to minimize animal suffering.

### 2.2. Viruses

A virulent strain of DHAV-3 was isolated from a clinically diseased Cherry Valley duckling in Sichuan Province (southwestern China) by propagating 10 times with the limiting dilution method in embryonated duck eggs via the allantoic cavity route. Unfortunately, the parental strain was unable to adapt to the chicken embryos, consistent with a previous report [20]. Thus, embryonated duck eggs were used for embryo adaptation. In brief, 8-day-old embryonated duck eggs (*n* = 5 per group) were inoculated with serial dilutions (10^–3^, 10^−4^, and 10^−5^ fold) of the virus suspensions via the allantoic cavity route and incubated at 37 °C. Embryos that died within 24 h post-infection (hpi) were abandoned. Subsequently, the allantoic fluid of the dead embryos at the lowest dilution of the virus was harvested at 72 hpi and used for the second passage. The embryo-adapted strain was propagated in antibody-negative embryonated duck eggs for 60 consecutive passages.

### 2.3. RNA Isolation and Complementary DNA (cDNA) Synthesis

Total RNA was extracted from the allantoic fluids or liver tissues using RNAiso Plus total RNA extraction reagent (TaKaRa Biotechnology (Dalian) Co., Ltd., Dalian, China). Subsequently, cDNA was produced using the PrimeScript RT Reagent Kit with gDNA Eraser (TaKaRa Biotechnology (Dalian) Co., Ltd.). The mRNA expression levels of the target genes were measured using TB Green Premix Ex Taq Master Mix or the One-Step PrimeScript RT-PCR Kit (TaKaRa Biotechnology (Dalian) Co., Ltd.), as described previously [21,22]. All samples were screened in triplicate.

### 2.4. Complete Genome Sequencing and Comparisons

The complete genome sequences of the virulent parental strain and related embryo-adapted strain were determined in accordance with a previously described method [23,24]. Comparative genomic analysis was performed using Molecular Evolutionary Genetics Analysis (MEGA) software, version X [25].

### 2.5. Replication Capacity of the Strains in Embryonated Duck Eggs

To determine the replication capacity of the virulent parental strain and related embryo-adapted strain in embryonated duck eggs, viral titers in the allantoic fluid were determined following procedures described previously [26]. Firstly, the RNA loads of the original virus-containing allantoic fluids were calculated quantitative real-time (qRT)-PCR analyses. Subsequently, the virus-containing allantoic fluids were subjected to two dilutions in PBS containing 10^3^ copies/μL and 10^5^ copies/μL viruses, respectively. A total of 200 μL of each strain at different dilutions was inoculated in 9-day-old embryonated duck eggs (*n* = 3). The eggs then incubated in an egg incubator at 37 °C. Embryos that died within 24 hpi were abandoned. The allantoic fluid of the dead embryos within 24–96 hpi was harvested. The virus titers of different strains were determined by qRT-PCR assay.

### 2.6. In Vivo Virus Infection

A total of 120 7-day-old ducklings were randomly assigned to one of three groups (*n* = 40). Each group was separated into two subgroups: 20 for sampling and 20 for clinical observation. Of the three groups, the infection groups were inoculated with 200 μL of either the virulent parental strain or the embryo-adapted strain containing 10^5.0^ copies/μL via the intramuscular route in the left leg, whereas the uninfected group was injected with the same volume of sterile phosphate-buffered saline (PBS, pH 7.4) via the same route to serve as a negative control. Post-challenge, the ducklings assigned to different groups were housed separately. The inoculated ducklings were monitored daily for clinical signs of infection and mortality. Meanwhile, three ducklings from each group were euthanized and necropsied at 12, 24, 36, 48, 72, and 120 hpi, respectively. The liver tissues were collected and divided into two portions: one for the determination of the viral RNA load and expression of innate immune genes by qRT-PCR analyses, whereas the second was fixed in neutral formalin for histopathological examination.

#### 2.6.1. Histopathological Analysis

The presence of microscopic lesions in the liver tissues of the ducklings was determined according to a previously described method [21]. The formalin-fixed, paraffin-embedded tissues were stained with hematoxylin and eosin to identify histopathological changes under a light microscope at 400× magnification.

#### 2.6.2. Viral Proliferation

To compare the replication capacities of the virulent and attenuated strains, the growth kinetics of both strains in the livers of ducklings were measured using a previously developed qRT-PCR assay [26]. Samples were recovered at the indicated time points.

#### 2.6.3. Innate Immune Gene Expression Analysis

The expression patterns of innate immune genes in duckling livers were determined by qRT-PCR analysis with the use of specific primers (Table 1) for the detection of β-actin, PRRs (TLR3, TLR7, MDA5, RIG-1, and NLRP3), IFNs (IFN-α, IFN-β, and IFN-γ), IL-1β, IL-2, IL-4, IL-6, and IL-10, and TNF-α. The expression levels of these genes were calculated using the comparative 2^−ΔΔCt^ method with β-actin as an endogenous control.

### 2.7. Statistical Analysis

All data are presented as the mean ± standard deviation (SD) and analyzed using GraphPad Prism 8 software (GraphPad Software, Inc., La Jolla, CA, USA). The Student’s *t*-test was used as indicated in each experiment and figure. A probability (*p*) value of <0.05 was considered statistically significant [* *p* < 0.05 (significant), ** *p* < 0.01 (highly significant), and *** *p* < 0.001 (very highly significant)].

## 3. Results

### 3.1. Two Mutations to the VP0 and the 2C Proteins are Involved in Embryo Adaptation of DHAV-3 

**Figure 1:**
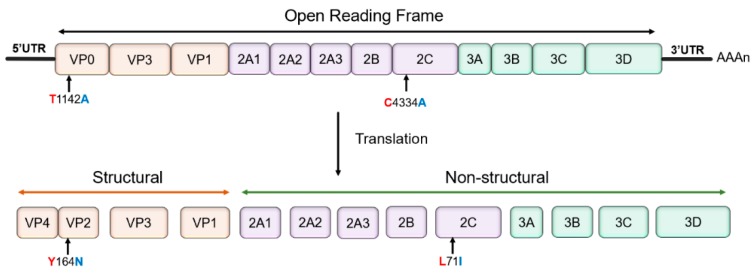
A schematic representation of the duck hepatitis A virus (DHAV)-3 genome. Comparative genomic analysis showed that there were two nucleotide substitutions (T1142A and C4334A) during the adaptation process, which led to two amino acid mutations in the VP0 (at residue 164) and 2C (at residue 71) proteins, respectively.

In this study, the virulent DHAV-3 strain was propagated in embryonated eggs to generate an embryo-adapted strain. The complete genome sequences of the parental and embryo-adapted strains were deposited into the GenBank database under the accession numbers MH752740 and MH752742, respectively. The genome sequences of the virulent and attenuated strains were aligned and analyzed using MEGA X software. Comparisons of the full-length genome sequences revealed two amino acid changes in the open reading frame, but no deletion/insertion mutations (Figure 1). After serial embryo-passages, there were two nucleotide substitutions (T1142A and C4334A) during the adaptation process, which led to two amino acid substitutions Y164N in the VP0 protein and L71I in 2C protein, respectively. Compared with the available complete genome sequences of DHAV-3 isolates retrieved from the GenBank, the amino acid substitution Y164N in the VP0 protein is unique to a majority of other DHAV-3 virulent strains (Y164), except GD strain (N164, GenBank: GQ122332.1,). Moreover, the amino acid substitution L71I in 2C protein is unique to all other DHAV-3 virulent strains (L71).

### 3.2. The Replication Capacity of the Strains in Embryonated Duck Eggs

**Figure 2:**
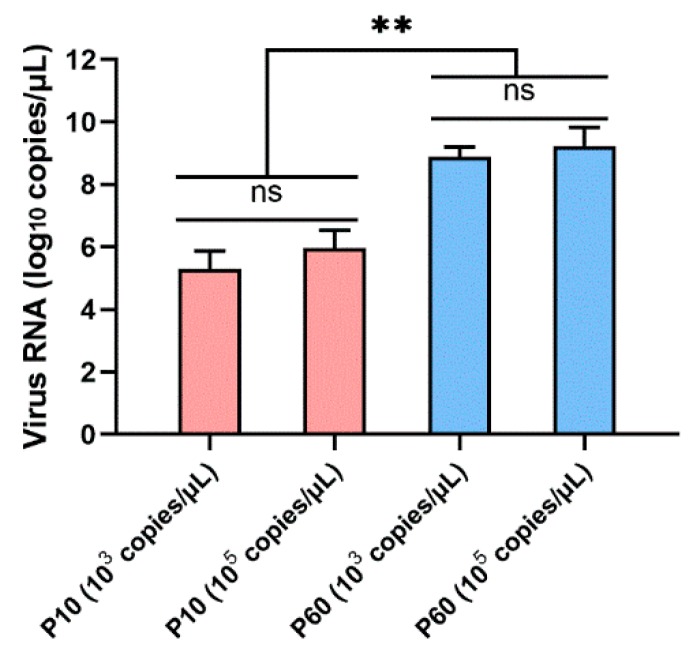
Viral titers of the virulent parental DHAV-3 strain and related embryo-adapted strain in the allantoic fluid of ducklings. The relative viral RNA load in the allantoic fluid was measured using qRT-PCR analyses. Each bar represents the mean ± SD (*n* = 3). The Student’s *t*-test was used to calculate the *p* values and 95% confidence levels. ** *p* < 0.01.

To analyze the replication capacity of the virulent parental strain and related embryo-adapted strain in embryonated duck eggs, viral titers in the allantoic fluid were assessed by qRT-PCR assay (Figure 2). The viral RNA load in the allantoic fluid of embryonated duck eggs infected with embryo-adapted strain was significantly higher than that infected with the virulent parental strain (*p* < 0.01). No significant difference was observed between the viral titers inoculated with different dilutions.

### 3.3. Clinical Signs and Gross Pathology of Ducklings Inoculated with the Virulent DHAV-3 Strain and Related Embryo-Adapted Strain

**Figure 3:**
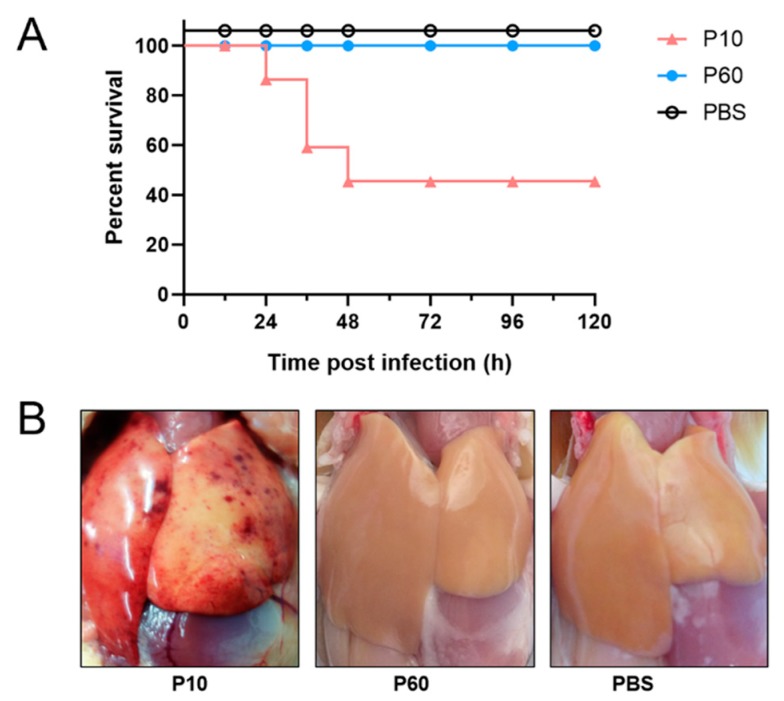
(**A**) Survival rate of ducklings inoculated with the virulent DHAV-3 strain, related embryo-adapted strain, or phosphate-buffered saline (PBS). Ducklings were inoculated through the intramuscular route. (**B**) Gross pathology of the liver tissues of ducklings after viral infection. At 36 hpi, the livers of the diseased ducklings were harvested for gross pathological assessment. The liver tissues of ducklings inoculated with the virulent strain exhibited obvious gross lesions, with punctate hemorrhaging and ecchymosis, whereas the liver tissues of the ducklings inoculated with the attenuated strain or PBS had no obvious lesions.

To further evaluate the pathogenicity of the virulent DHAV-3 strain and related embryo-adapted strain, 7-day-old ducklings were inoculated with 200 μL (10^5.0^ copies/μL) of virus per duckling via the intramuscular route. During the observation period, ducklings inoculated with the virulent strain developed typical clinical symptoms (i.e., lethargy, ataxia, and opisthotonos), which resulted in a morbidity rate of 80% and a mortality rate of 60% at 120 hpi (Figure 3A). On post-infection day 1, ducklings in the virulent parental strain group developed clinical signs of infection, which resulted in the death of three ducklings at 24 hpi, six at 36 hpi, and three at 48 hpi. Notably, the attenuated strain did not cause clinical disease during the observation period.

At necropsy, the liver tissues of the diseased ducklings inoculated with the virulent strain exhibited obvious gross lesions with punctate hemorrhaging and ecchymosis (Figure 3B). Meanwhile, the spleens of several ducklings were enlarged. In contrast, none of the ducklings inoculated with the attenuated strain or PBS had obvious lesions. Thus, acute liver injury seemed to be the main cause of death. Together, these observations indicate that the serially embryo-passaged virus was attenuated in vivo.

### 3.4. Histopathologic Lesions of the Liver Tissues of Ducklings Inoculated with the Virulent DHAV-3 Strain and Related Embryo-Adapted Strain

**Figure 4:**
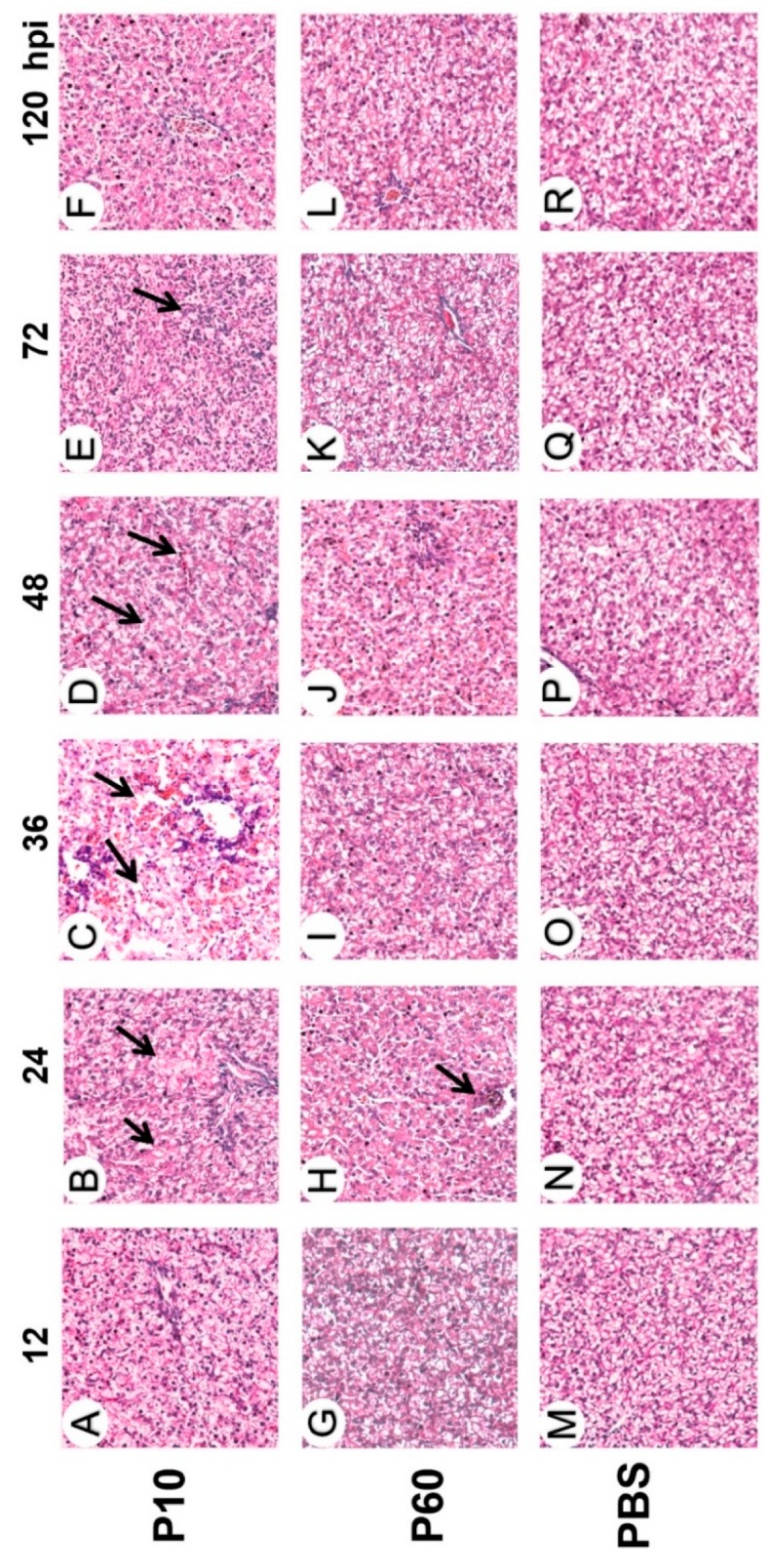
Histopathologic lesions detected in the liver tissues of ducklings inoculated with the virulent DHAV-3 strain (**A–F**), related embryo-adapted strain (**G–L**), and PBS (**M–R**). The livers had vacuolation and necrosis of hepatocytes (black arrows in **B** and **C**), a large number of infiltrating inflammatory cells, and multifocal confluent coagulative necrosis (black arrows in **D** and **E**).

As presented in Figure 4, the liver tissues of ducklings inoculated with the virulent strain exhibited severe histopathological changes with microscopic lesions and damaged parenchyma cells. Notably, liver tissues from the ducklings inoculated with the virulent strain showed extensive cell death, along with destruction, severe vacuolization, and necrosis of hepatocytes at 24 to 48 hpi (Figure 4A–F). On the contrary, the livers of ducklings inoculated with the attenuated strain had significantly fewer histopathological lesions, as compared with those infected with the virulent strain (Figure 4G–L). Meanwhile, the livers of ducklings inoculated with the attenuated strain exhibited slight histological changes at 24 hpi (Figure 4H). There were no obvious histological changes in the livers of ducklings inoculated with the attenuated strain or negative control (PBS) at any time point (Figure 4M–R). Together, these observations indicate that the attenuated strain caused a non-cytopathic infection.

### 3.5. Replication Capacity of the Attenuated Strain Was Restricted in the Livers of the Inoculated Ducklings

**Figure 5:**
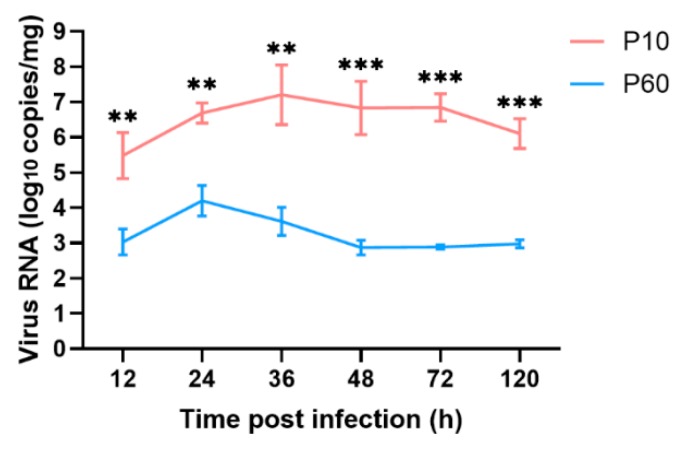
In vitro growth kinetics of the virulent strain and its embryo-passaged strain of DHAV-3 in the liver tissues of ducklings. The relative viral RNA load in liver tissues was measured by qRT-PCR. Each bar represents the mean ± SD (*n* = 3). The Student’s *t*-test was used to calculate the *p* values and 95% confidence levels. ** *p* < 0.01, and *** *p* < 0.001 (***) vs. the control group.

At the indicated times post-infection, the liver tissues of ducklings (*n* = 3 per group) were collected for the detection of virus titers, which reflect the copy numbers of the viral RNA genome. Virus titers were quantified using a previously developed qRT-PCR assay [26]. The results showed that all liver tissues of the infected ducklings were positive for DHAV-3 during the experimental period, whereas those in the control group were negative. Notably, the titer of the attenuated strain was significantly lower in the liver tissues of the inoculated ducklings, as compared with the virulent parental strain, at all time points (Figure 5), indicating decreased replication capacity of the attenuated strain (*p* < 0.01).

The viral RNA load in the livers of the ducklings infected with the virulent strain reached the highest level at 36 hpi (10^7.2^ copies/mg) and then gradually decreased over time from 36 to 120 hpi (10^6.8^ to 10^6.1^ copies/mg, respectively). In contrast, the viral proliferation of the attenuated strain was effectively inhibited after 24 hpi (10^4.2^ copies/mg). Also, the viral genome copy number of the attenuated strain in the livers was maintained at a lower level over time from 48 to 120 hpi (10^2.6^ to 10^3.1^ copies/mg, respectively). These data demonstrate that the replication capacity of the attenuated strain was limited in the liver tissues of the inoculated ducklings.

### 3.6. The Attenuated Strain Lead to the Activation of Unique Innate Immune Genes in the Liver Tissues of the Inoculated Ducklings

**Figure 6:**
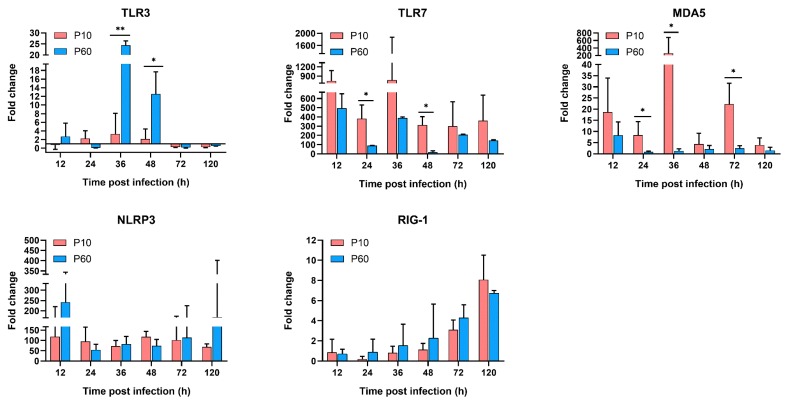
Dynamic activation of pattern recognition receptors (PRRs) genes in the livers of inoculated ducklings. Expression levels of these genes were detected by qRT-PCR analyses and analyzed using the 2^-ΔΔCt^ method. Each bar represents the mean ± SD (*n* = 3). The Student’s *t*-test was used to calculate the *p* values and 95% confidence levels. * *p* < 0.05, ** *p* < 0.01.

**Figure 7:**
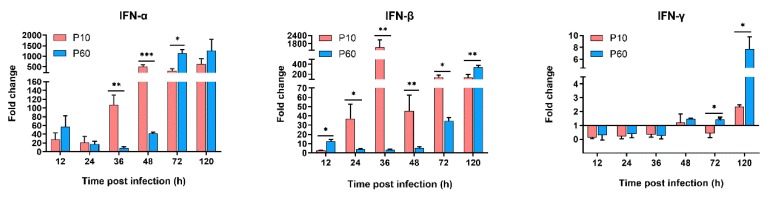
Dynamic activation of IFNs genes in the livers of inoculated ducklings. Expression levels of these genes were detected by qRT-PCR analyses and analyzed using the 2^-ΔΔCt^ method. Each bar represents the mean ± SD (*n* = 3). The Student’s *t*-test was used to calculate the *p* values and 95% confidence levels. * *p* < 0.05, ** *p* < 0.01, and *** *p* < 0.001 (***) vs. the control group.

**Figure 8:**
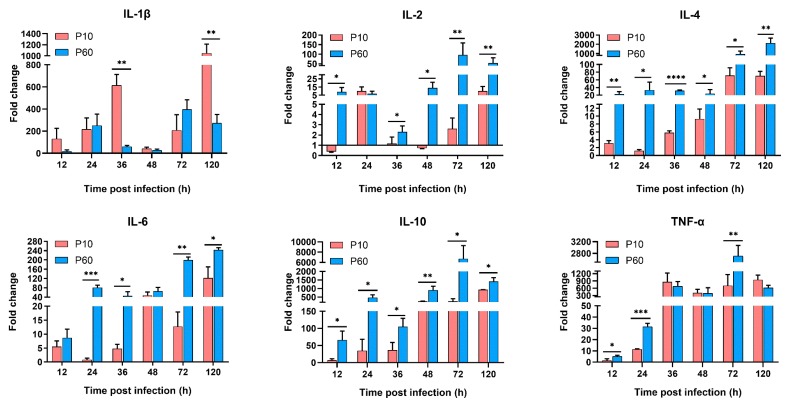
Dynamic activation of proinflammatory cytokines and anti-inflammatory cytokines genes in the livers of inoculated ducklings. Expression levels of these genes were detected by qRT-PCR analyses and analyzed using the 2^-ΔΔCt^ method. Each bar represents the mean ± SD (*n* = 3). The Student’s *t*-test was used to calculate the *p* values and 95% confidence levels. * *p* < 0.05, ** *p* < 0.01, and *** *p* < 0.001 (***) vs. the control group.

To identify the innate immune genes essential for DHAV-3 invasion, the mRNA expression levels of PRRs (TLR3, TLR7, MDA5, RIG-1, and NLRP3), IFNs (IFN-α, IFN-β, and IFN-γ), proinflammatory cytokines (IL-1β, IL-2, IL-6, and TNF-α), and anti-inflammatory cytokines (IL-4 and IL-10) in the liver tissues of the inoculated ducklings were examined and compared with those of the negative control group. As expected, the virulent parental and attenuated strains differentially regulated the mRNA expression levels of the innate immune genes in the liver tissues of ducklings. As shown in Figure 6, Figure 7 and Figure 8, the expression levels of most of the tested genes were upregulated in the viral infection groups during the experimental period.

#### 3.6.1. The PRRs Expression Analysis

Among the PRRs expressed in liver tissues (Figure 6), TLR7 activation is essential for the IFN response to both DHAV-1 and DHAV-3 infection [15,16,27]. Notably, the mRNA levels of TLR7 were higher following infection of the virulent strain, as compared with the attenuated strain. In this study, the mRNA levels of TLR7 and NLRP3 were significantly increased in both infection groups (*p* < 0.05). The expression levels of TLR7 were similar in both groups. However, the level of TLR-7 was higher in the tissues of the ducklings infected with the virulent strain at all time points, which may be related to the higher viral RNA load. Consistent with the findings of a recent study, the mRNA levels of TLR3 were very low in the group infected with the virulent strain (up to 8.8-fold). However, the level of TLR3 in the group infected with the attenuated strain had transiently increased at 36 and 48 hpi (up to 25.8- and 17.8-fold, respectively). In contrast, the mRNA expression level of MDA5 was significantly higher following infection with the virulent strain at 24, 36, and 72 hpi (*p* < 0.05). Furthermore, the mRNA levels of RIG-1 increased gradually in a time-dependent manner, without significant differences between these two strains.

#### 3.6.2. The IFNs Expression Analysis

It has been reported that infection with pathogenic DHAV-3 could increase the mRNA expression levels of IFN-α, IFN-β, and IFN-γ [15]. Similarly, in the present study, infection with both the virulent and non-virulent strains triggered far greater IFN production at the end of the experiment (Figure 7). Specifically, the virulent strain caused acute induction of IFN-α and IFN-β mRNA expression at the early stage of infection (12–48 hpi) with a moderate increase in IFN-γ expression at the end of the experiment (120 hpi). In contrast, the attenuated strain only moderately enhanced IFN-α and IFN-β mRNA expression at the early stage of infection (12–48 hpi) with moderately higher levels of IFN-α, IFN-β, and IFN-γ at the late stage of infection (72–120 hpi). However, infection with the attenuated strain resulted in higher levels of IFN-β at 12 and 120 hpi, as compared with the virulent strain. IFN-β expression began to markedly increase in the group infected with the virulent strain at 24 hpi, then peaked at 36 hpi. Also, IFN-β expression was maintained at a higher level at 24–72 hpi (*p* < 0.05), as compared with the group infected with the virulent strain, which was consistent with the histopathological lesions observed in the liver tissues.

Together, these results suggest that excessive IFN-β production in the early stages of infection may play a role in the pathogenicity of DHAV-3. Furthermore, IFN-γ mRNA expression was significantly increased in the group infected with the attenuated strain at the late stage of infection (72–120 hpi, *p* < 0.05), indicating that the attenuated strain could induce a stronger adaptive immune response.

#### 3.6.3. The Proinflammatory Cytokines and Anti-Inflammatory Cytokines Expression Analysis

Among the tested proinflammatory and anti-inflammatory cytokines (Figure 8), there were significant differences in the mRNA expression levels of IL-2, IL-4, IL-6, and IL-10 following infection with the virulent vs. the attenuated strain (*p* < 0.05). Notably, the expression levels of the anti-inflammatory cytokines (IL-4 and IL-10) were significantly upregulated in the tissues of ducklings infected with the attenuated strain at all indicated time points (*p* < 0.05). Besides, significant upregulation of the proinflammatory cytokines (IL-2 and IL-6) was observed in the virulent strain group at multiple time points. These results indicated that infection with the attenuated strain could induce higher expression of anti-inflammatory cytokines to reduce inflammation, restore liver homeostasis, and avoid immune-mediated liver injury. In addition, the IL-1β expression patterns were irregular in both infection groups but were significantly upregulated following infection with the virulent strain at 36 and 120 hpi (*p* < 0.05). Furthermore, the expression level of TNF-α continuously increased from before 36 hpi and then was maintained at significantly high levels until the end of the experiment. Based on these results, we hypothesized that the attenuated strain could reactivate a well-regulated innate immune response without immune-mediated injury, which may contribute to the attenuation of liver injury and limit disease.

## 4. Discussion

DHAV is the major etiological agent responsible for DVH outbreaks worldwide. In vivo studies have demonstrated that infections with virulent and attenuated strains of DHAV-1 activate different innate immune responses in ducklings [21,28,29,30,31]. Especially, the pathogenic DHAV-1 strain causes more significant systemic infections, characterized by severe hepatic injury, in young ducklings aged <3 weeks [27,28]. The results of a recent study suggest that excessive cytokine production (“cytokine storm”) in the liver tissues may be associated with severe pathology of ducklings infected with the virulent DHAV-1 strain [21]. In addition, as a crucial countermeasure for the prevention and control of DHAV-1 infection, natural products and their derivatives were reported to lessen the degree of hepatic injury [32,33,34,35]. Furthermore, it has been recently reported that the differences in immune responses correlate with viral attenuation [21]. After serial passages in embryonated chicken eggs, the attenuated strain of DHAV-1 was able to damage the type I IFN response and thereby promote viral propagation [36].

Although recent studies have reported that classical DHAV-1 and DHAV-3 have a similar pathogenicity, and both can induce a strong host immune response with enormous production of IFNs and inflammatory cytokines [15,16,37], no study has yet to investigate the pathophysiological and immunological mechanisms of DHAV-3 isolates with varying degrees of pathogenicity. Furthermore, a recently developed chicken embryo-adapted DHAV-3 vaccine was shown to be efficacious in clinical trials conducted in South Korea [38,39]. However, the mechanisms underlying viral attenuation remain unclear, and the reason for the difference in the innate immune responses between the virulent and attenuated DHAV-3 strains remains unknown.

In this study, a virulent DHAV-3 strain was serially passaged 60 times in duck embryos to obtain an embryo-adapted strain. To determine the strain-specific molecular contributions to embryo adaptation, the complete genomes of these two strains were sequenced. There were just two amino acid differences between the virulent parental and attenuated strains, suggesting that the DHAV-3 strain has high genetic stability over serial passages. It seems that these two mutations (Y164N and L71I) determine the attenuation phenotype. To determine whether these two mutations are directly involved in the pathogenesis of strain DHAV-3, a reverse genetic system was used to construct different mutants. Furthermore, the contributions of these mutations to the pathogenicity of other DHAV-3 strains are being investigated to elucidate the molecular basis underlying viral attenuation.

As a member of picornaviruses, DHAV comprises a capsid, surrounding the viral genome. The viral genome is a single standard positive sense RNA which contains a single open reading frame (ORF) flanked by 5′ and 3′ untranslated regions (5′-UTR and 3′-UTR). The polyprotein encoded by the ORF is processed into structural (VP0, VP3, and VP1) and nonstructural proteins (2A, 2B, 2C, 3A, 3B, 3C, and 3D). The capsid, which is structurally similar to several members of the picornaviruses, comprises 60 copies each of the three different structural polypeptides VP0 (uncleaved precursor of VP2 and VP4), VP1, and VP3. The viral genome is expected to be highly condensed in mature virus particles, composed of VP1-VP3 located on the outer surface, and some part of VP0 located in the interior of the capsid.

Capsid proteins could interact with host factors to subvert various cellular processes for viral infection, according to the characteristics of the virus. Evidence suggests that capsid-receptor interactions play decisive roles in viral attachment, internalization, and entry, thus determining cell types, tissues, and species tropisms [40,41]. Specifically, amino acid substitutions in the VP2 can influence the replicative ability and virulence of various picornaviruses, such as EV71 [42,43], foot-and-mouth disease virus [44,45,46,47,48], Coxsackievirus B3 [49,50,51], and Theiler’s murine encephalomyelitis viruses [52,53]. Thus, special consideration should be given to the VP2 protein in research on structure-function relationships and in vaccine manufacture. We speculated that Y164N substitution in 2C protein of DHAV-3 might change the viral tropisms, which may be related to increased viral replication capacity in embryonated duck eggs and reduced replication capacity in ducklings. Moreover, an adaptive mutation F164S of the VP0 protein had occurred during the chicken embryo attenuation process of the virulent DHAV-3 strain, as determined in a study conducted in South Korea [38]. Further investigations are required to study whether the same attenuation mechanism exists for these viruses.

It has been revealed that the 2C protein of picornaviruses is essential for viral replication. It could mediate viral RNA synthesis [54,55], mediate virus encapsidation [56,57], induce the formation of membrane vesicles [58], and suppress the activation of the innate immune response [59,60,61]. Notably, previous studies have reported that mutations in 2C protein contribute to adaptation to cell culture and also were responsible for the attenuated phenotype of hepatitis A virus [62,63,64,65]. We speculated that L71I substitution in 2C protein of DHAV-3 might change the viral RNA synthesis function and result in different effects on the viral replication capacity in the embryonated duck eggs and ducklings.

In the present study, the replication capacity, pathogenicity, and capacity to induce an innate immune response of the virulent parental and attenuated strains were investigated. Inoculations of 7-day-old ducklings with the virulent parental strain, related embryo-adapted strain, and PBS were performed intramuscularly. The virulent parental strain caused severe clinical signs and gross lesions, which resulted in high mortality. The microscopic lesions of the liver tissues were consistent with the gross lesions, whereas no obvious histological changes were caused by the attenuated strain, as compared with the negative control group. In addition, the titer of the attenuated strain, as compared with that of the virulent parental strain, was significantly lower in the livers of the inoculated ducklings at all time points. Meanwhile, consistent with the gross lesions described above, the abundance of microscopic lesions had increased with the increased proliferation of the virulent strain. In agreement with the reduced viral RNA load, damage to the liver parenchyma was significantly decreased in the ducklings infected with the attenuated virus, suggesting that the replication capacity of the attenuated strain of DHAV-3 was reduced and had a non-cytopathic effect in the liver tissues of the inoculated ducklings.

Recent data suggest that acute DHAV-3 infection involves excessive cytokine production, which leads to the development of acute liver injury and death of the infected ducklings [15,66]. Similarly, in the current study, the upregulation of the expression levels of innate immune genes was observed in ducklings infected with DHAV-3. Among the PRRs, the mRNA expression levels of TLR3, TLR7, and MDA5 were upregulated to different degrees in the groups infected with the virulent vs. attenuated strain. We speculate that the upregulated expression of MDA5 and TLR7 in the group infected with the virulent strain lead to the upregulation of IFN-α and IFN-β mRNA levels. In addition, the mRNA level of TLR3 was maintained at a very low level in the virulent strain group, consistent with the findings of previous studies [15,66]. However, the TLR3 mRNA level in the group infected with the attenuated strain dramatically increased at some time points. Coincidently, a previous study revealed that TLR3 could stimulate the production of IL-10 to ameliorate alcoholic liver injury [67]. We speculate that the upregulation of TLR3 may play a similar role in response to infection with the attenuated DHAV-3 strain.

Simultaneously, the dramatically increased expression of IFN-α and IFN-β in the livers of ducklings inoculated with the virulent strain in the early stage of infection are in line with previous studies [15,66]. Notably, simultaneous peaks in the viral RNA load and upregulated expression of IFN-β have been observed in ducklings inoculated with the virulent strain at 36 hpi. In contrast, ducklings inoculated with the attenuated strain showed no significant change in the expression levels of IFN-α and IFN-β during the early stage of infection. However, the mRNA levels of IFN-α, IFN-β, and IFN-γ were higher in the group infected with the attenuated strain, as compared with the virulent strain, at the end of the experiment. Dysregulated type I IFN is related to inflammatory and cytotoxicity responses [68,69,70], especially IFN-β-induced liver injury via immune-mediated hepatocellular damage [71]. Therefore, further studies are required to determine whether dysregulated type I IFN plays a critical role in the pathogenicity of DHAV-3 infection.

Interestingly, the results of the present study confirmed that the attenuated strain of DHAV-3 could induce higher expression levels of IL-2, IL-4, IL-6, and IL-10 in the liver tissues of infected ducklings. Notably, the release of anti-inflammatory cytokines (IL-4 and IL-10) was significantly increased in the liver tissues of ducklings infected with the attenuated strain at all indicated time points. IL-10 is mostly regarded as an anti-inflammatory cytokine that plays a central role in a variety of human diseases through the maintenance of immune homeostasis [72]. Previous studies have revealed that Il-10 can prevent hepatic steatosis and other metabolic abnormalities with anti-inflammatory, immunosuppressive, tolerogenic, and neuroprotective properties [73,74,75]. The mechanism employed by the attenuated strain of DHAV-3 to stimulate anti-inflammatory cytokines remains unclear. In addition, the expression mRNA levels of proinflammatory cytokines (IL-2 and IL-6) were significantly upregulated in the virulent strain group at multiple time points. The effect of IL-2 and IL-6 on hepatic injury is unclear due to conflicting reports [76]. For example, IL-6, as a proinflammatory cytokine, can also protect the liver from CD8+ T cell-mediated injury [77]. Nonetheless, further studies are required to elucidate the specific mechanisms of these pleiotropic cytokines in the pathogenicity of DHAV-3 infection.

## 5. Conclusions

Altogether, this study assessed the genetic characteristics and pathogenesis of the virulent DHAV-3 strain and related embryo-adapted strain. The results demonstrated that two amino acid substitutions (Y164N in VP0 protein and L71I in 2C protein) introduced during the adaptation process. In addition, we confirmed the replication capacity of the virulent parental strain and related embryo-adapted strain were significantly different in embryonated duck eggs and ducklings. We speculated that the embryo adaptation of DHAV-3 reduced the replication capacity in duckling and ultimately lead to the attenuation phenotype. In addition, different innate immune responses in the liver of ducklings were at least partly responsible for the differential infectivity phenotype. These results extend our understanding of the viral replication and immune regulation in the virulence of DHAV-3, as well as candidate amino acid substitutions related to viral attenuation. A better understanding of the genetics and pathogenesis of DHAV-3 may aid in the development of new vaccines and the implementation of immunization strategies.

## Figures and Tables

**Table 1:** Sequences of the primers for qRT-PCR analysis.

Target Gene	Sense Primer (5’-3’)	Antisense Primer (5’-3’)
β-actin	TACGCCAACACGGTGCTG	GATTCATCATACTCCTGCTTG
TLR-3	AACACTCCGCCTAAGTATCAT	CTATCCTCCACCCTTCAAAA
TLR-7	CCTTTCCCAGAGAGCATTCA	TCAAGAAATATCAAGATAATCACATCA
MDA5	CTGCCCGCTACTTGAACTCCA	GCACCATCTCTGTTCCCACGA
RIG-1	GCGTACCGCTATAACCCACA	CCTTGCTGGTTTTGAACGC
NLRP-3	CATCCCAGTGAAGCGTTGA	GCCATCTGGTCGTATAGCG
IFN-α	TCCACCTCCTCCAACACCTC	TGGGAAGCAGCGCTCGAG
IFN-β	CCTCAACCAGATCCAGCATT	GGATGAGGCTGTGAGAGGAG
IFN-γ	GCTGATGGCAATCCTGTTTT	GGATTTTCAAGCCAGTCAGC
IL-1β	TCGACATCAACCAGAAGTGC	GAGCTTGTAGCCCTTGATGC
IL-2	TCCCTGAATTTCGCCAAG	AGCGGACAGCAAGTTAGGTAGC
IL-4	TACCTCAACTTGCTGCACATC	GCTACTCGTTGGAGGGTTCT
IL-6	TTCGACGAGGAGAAATGCTT	CCTTATCGTCGTTGCCAGAT
IL-10	AGCAGCGAGCACCACCA	TGCCGTTCTCGTTCATCTTT
TNF-α	TTTTATGACCGCCCAGTT	TAGGCAGAGGCCACCA
